# Hapln1a Is Required for Connexin43-Dependent Growth and Patterning in the Regenerating Fin Skeleton

**DOI:** 10.1371/journal.pone.0088574

**Published:** 2014-02-12

**Authors:** Jayalakshmi Govindan, M. Kathryn Iovine

**Affiliations:** Department of Biological Sciences, Lehigh University, Bethlehem, Pennsylvania, United States of America; Heart Science Centre, Imperial College London, United Kingdom

## Abstract

Cell–cell communication, facilitating the exchange of small metabolites, ions and second messengers, takes place via aqueous proteinaceous channels called gap junctions. Connexins (cx) are the subunits of a gap junction channel. Mutations in zebrafish *cx43* produces the *short fin* (*sof ^b123^*) phenotype and is characterized by short fins due to reduced segment length of the bony fin rays and reduced cell proliferation. Previously established results from our lab demonstrate that Cx43 plays a dual role regulating both cell proliferation (growth) and joint formation (patterning) during the process of skeletal morphogenesis. In this study, we show that Hapln1a (Hyaluronan and Proteoglycan Link Protein 1a) functions downstream of *cx43*. Hapln1a belongs to the family of link proteins that play an important role in stabilizing the ECM by linking the aggregates of hyaluronan and proteoglycans. We validated that *hapln1a* is expressed downstream of *cx43* by in situ hybridization and quantitative RT-PCR methods. Moreover, in situ hybridization at different time points revealed that *hapln1a* expression peaks at 3 days post amputation. Expression of *hapln1a* is located in the medial mesenchyme and the in the lateral skeletal precursor cells. Furthermore, morpholino mediated knock-down of *hapln1a* resulted in reduced fin regenerate length, reduced bony segment length and reduced cell proliferation, recapitulating all the phenotypes of *cx43* knock-down. Moreover, Hyaluronic Acid (HA) levels are dramatically reduced in *hapln1a* knock-down fins, attesting the importance of Hapln1a in stabilizing the ECM. Attempts to place *hapln1a* in our previously defined *cx43*–*sema3d* pathway suggest that *hapln1a* functions in a parallel genetic pathway. Collectively, our data suggest that Cx43 mediates independent Sema3d and Hapln1a pathways in order to coordinate skeletal growth and patterning.

## Introduction

Gap junctions play a critical role in coupling tissue function and they have long been hypothesized to play a role in the maintenance of homeostasis, morphogenesis, cell differentiation, growth control and the process of skeletogenesis in multicellular organisms [1]. Gap junctions are proteinaceous channels formed by the docking of two connexons between neighboring cells, and they mediate the exchange of low molecular weight metabolites (<1000 Da), ions and second messengers between the contacting cells [2]. Each connexon or hemichannel is made up of six connexins, each protein containing a four pass transmembrane domain. The syndrome oculodentodigital dysplasia (ODDD), characterized by abnormalities in craniofacial elements, limbs and dentition, has been linked to missense mutations in the *GJA1* gene locus in humans [3]. At least 24 separate point mutations in *GJA1*, which codes for Connexin43 (Cx43), have been identified in patients with ODDD [3]–[5]. The *CX43* knock out (*CX43^−/−^*) mouse dies perinatally because of cardiac malformations [6], [7]. Similarly, targeted gene knock-down of *cx43* results in embryonic heart defects in zebrafish, signifying the essential role of *cx43* during development [8]. The skeletal defects seen in the *CX43^−/−^* KO mouse model exhibited hypomineralization of craniofacial bones and severely delayed ossification of the appendicular skeleton [9]. Moreover, the ODDD phenotype is similar to a set of craniofacial abnormalities observed in the targeted *CX43* knock-down chick model [10], [11]. In zebrafish, a homozygous mutation in *cx43* causes the *short fin* phenotype, characterized by shorter tail fins due to defects in the fin skeleton. The mechanism by which *CX43* based mutations cause skeletal defect phenotypes is largely unknown. However, it is apparent that the function of Cx43 in the vertebrate skeleton is conserved.

We utilize the zebrafish *short fin* mutant (*sof ^b123^*) to address the role of Cx43 during skeletal development. Our lab has found that the mutation in zebrafish *cx43* gene causes the *short fin* (*sof ^b123^*) phenotypes characterized by short bony fin ray segments, short fins, and reduced cell proliferation. The *sof ^b123^* mutant exhibits reduced levels of *cx43* mRNA and Cx43 protein, without a lesion in the coding sequence [8]. Moreover, three additional alleles that cause missense mutations also resulted in reduced gap junctional intercellular communication (GJIC), short segments, and reduced cell proliferation [12]. Furthermore, morpholino mediated knock-down of Cx43 in wild type zebrafish completely recapitulate all the phenotypes produced by *sof* alleles [13]. In contrast to *sof ^b123^*, the *another long fin* (*alf ^dty86^*) mutant exhibits fin overgrowth and overlong segments due to stochastic joint failure [14]. These phenotypes are opposite those of *sof*, and we have shown that *alf ^dty86^* exhibits increased levels of *cx43* mRNA [15]. Indeed, *cx43* knock-down in *alf ^dty86^* rescues the segment length phenotype, suggesting that Cx43 over expression contributes to the *alf ^dty86^* phenotypes. (note that the mutation causing *alf ^dty86^* phenotypes is not located in the *cx43* gene [15]). We interpret the *cx43*-dependent loss of joints in *alf ^dty86^* and the premature joint formation (i.e. short segment) phenotype in *sof* to indicate that Cx43 suppresses joint formation. At the same time, Cx43 is positively associated with cell proliferation. Thus, Cx43 functions in more than one way, both positively influencing cell proliferation and negatively influencing joint formation, thereby concomitantly regulating bone growth and skeletal patterning during the process of fin regeneration.

An important, yet poorly understood question with respect to mutations in *connexin* genes in general is, how does GJIC impact tangible cellular events like cell division and differentiation? One hypothesis is that Cx43 based GJIC can influence gene expression patterns [16], [17]. Our lab exploited the availability of the two mutants, *sof ^b123^* and *alf ^dty86^*, in order to identify genes whose expression depends on Cx43. Thus, we utilized a novel microarray strategy to identify a set of candidate genes, which are both downregulated in *sof ^b123^* and upregulated in *alf ^dty86^*. The first gene validated from this microarray is *semaphorin3d (sema3d)* [18]. Here, we provide molecular and functional validation of another gene identified from the microarray analysis, *hapln1a* (*hyaluronan and proteoglycan link protein 1a*). In mouse and human, the orthologous protein Hapln1 has also been referred to as either cartilage link protein (Crtl1) or link protein (LP). The function of Hapln1 is to “link” hyaluronic acid (HA) with proteins termed proteoglycans (PG) in the extracellular matrix (ECM). Remarkably, the mouse knockout for *CRTL1* (aka *HAPLN1*) causes dwarfism, craniofacial abnormalities, and perinatal lethality in the mouse [19].

The ECM is a complex mixture of proteins and carbohydrates that forms a dense network surrounding cells. Little is known about the functional role of the ECM during zebrafish fin regeneration. Pharmacological treatments that alter the localization of PGs and collagen, as well as the expression levels of matrix metalloproteases, have been found to restrict outgrowth during fin regeneration [20], [21]. Even less is known about the particular role of the Hapln1a-based ECM, including HA, in the regenerating fin. HA is a large molecular weight carbohydrate polymer with a molecular mass up to 10^6^–10^7^ Da [22], [23]. The structure of the ECM is stabilized by Haplns by virtue of forming stable associations between HA and PGs [24]–[26]. Unsulfated glycosaminoglycans (such as HA) are prominent in the blastemas of late stage regenerating cichlid fins, although their function has not been elucidated [27]. Apart from contributing to the physicochemical properties, ECM turnover and remodeling are critical events during tissue injury and wound repair. HA is also known to regulate cell migration, proliferation, and differentiation through activation of HA-specific cell surface receptors [28]–[30]. This is the first study to evaluate the effects of destabilizing the Hapln1a-based ECM during zebrafish fin regeneration. We find that reduced Hapln1a causes skeletal growth and patterning defects during fin regeneration, perhaps via destabilization of HA. Furthermore, we find that *hapln1a* functions downstream of *cx43*, providing novel insights into how skeletal morphogenesis could be influenced by Cx43.

## Materials and Methods

### Statement on the ethical treatment of animals

This study was carried out in strict accordance with the recommendations in the Guide for the Care and Use of Laboratory Animals of the National Institutes of Health. The protocols used for this manuscript were approved by Lehigh's Institutional Animal Care and Use Committee (IACUC) (protocol identification #128, approved 11/14/2012). Lehigh University's Animal Welfare Assurance Number is A-3877-01. All experiments were performed to minimize pain and discomfort.

### Animal procedures

The following zebrafish (*Danio rerio*) strains were used in this study: wild-type C32, *sof ^b123^* [31] and *alf ^dty86^* [14]. Fish were maintained at a constant temperature of 25°C with 14 light: 10 dark photoperiod [32]. Fish were anaesthetized in 0.1% tricaine and caudal-fin amputations were performed at the 50% level using razor blades. Fin regeneration was then allowed to proceed until the desired time period and the regenerated fins were harvested from anaesthetized fish. Fins were fixed overnight in 4% paraformaldehyde (PFA) in PBS and dehydrated in 100% methanol at −20°C. Knockdown and whole mount in situ hybridization experiments were performed in triplicate with 5 fins per trial. For histochemistry on sectioned tissue, a minimum of 10 sections were evaluated from each of 3 different fins. For quantitative analyses, student's t-tests were completed to determine statistical significance (p<0.05).

### *In situ* hybridization on whole mount and cryosectioned fins

RNA probes were generated from PCR amplified linear DNA in which the reverse primer contained the T7 RNA polymerase binding site. The primers used in this study for ISH are summarized in Table 1. Digoxygenin (DIG) labeled RNA probes were synthesized using DIG labeling mix (Roche) and in situ hybridization on regenerated whole fins was carried out as described [18]. Briefly, for the whole mount method, fins stored in methanol were sequentially rehydrated with methanol/PBST and then treated with 5 µg/ml proteinase K for 45 min and re-fixed in 4%PFA in PBS for 20 min followed by extensive washes with PBST and finally the pre-hybridization process was carried out with HYB (50% formamide, 5X SSC, 10 mM citric acid, 0.1% Tween20, heparin and tRNA) at 65°C for 0.5–1 h. Hybridization with DIG labeled probes suspended in HYB was carried out overnight at 65°C followed by sequential washes with HYB/PBST and finally with PBST. Anti-DIG Fab (Roche) fragments were used at 1∶5000 dilution, followed by extensive PBST washes and short washes in staining buffer. Subsequently fins were transferred to a staining solution containing NBT and BCIP and allowed to develop under dark conditions until purple color was observed. The reaction was stopped by washing in PBST and fixing overnight with 4% PFA. Finally the fins were mounted on a glass slide and analyzed using a Nikon Eclipse 80i microscope. Pictures were taken using a digital Nikon camera.

**Table 1:** Primers and Morpholinos.

Gene	Primers for ISH	Primers for qRT-PCR	Morpholinos
***hapln1a***	F = GGTTCTCCGCTTGGCAGCG RT7 = **TAATACGACTCACTATAGGG**CCCATCCCTGCCTAAGACC	F = AACGACTATGGCACATACCGG R = AAGGTTGTACCGCCCCAAA	Splice MO = ATCAAAAGTGATTCTTACCTTGTGC ATG MO = GCCACAGAAAACAGAGCAATCATCT 5MM MO = GCCAGACAAAAGAGACCAATGATCT
***sema3d***	F = CGAAGTGTAGTACCATTTACG RT7 = **TAATACGACTCACTATAGGG**TATGAGGATCATATGTCC	F = TGGATGAGGAGAGAAGCCGAT R = GCAGGCCAGCTCAACTTTTT	MO = TGTCCGGCTCCCCTGCAGTCTTCAT 5MM = TGTGCCGCTGCCCTCCACTCTTCAT
***actin***		F = TTTTGCTGGAGATGATGCCC R = CCAACCATCACTCCCTGATGT	

The T7 RNA polymerase binding site is in bold in the reverse primers. F = Forward primer; RT7 = Reverse primer; MO = targeting morpholino; ATG MO = targets translation initiation; Splice MO = targets splicing event; 5MM = control morpholino with 5 mismatch pairs to target sequence. All primers and MOs are shown in the 5′-3′orientation.

For in situ on sections, fins were fixed overnight with 4% PFA in PBS after harvest. After a brief methanol wash, fins were dehydrated in 100% methanol and stored at −20°C until use. Before sectioning, fins were sequentially rehydrated in a methanol-PBS series of washes and then were embedded in 1.5% agarose/5% sucrose dissolved in PBS and equilibrated overnight in 30% sucrose. Fins were mounted in OCT and cryosectioned (15 µm sections) using a Reichertâ Jung 2800 Frigocut cryostat. Sections were collected on Superfrost Plus slides (Fisher) and allowed to air dry overnight at room temperature. Sections can be stored at −20°C for up to a year. Before use, the slides were brought to room temperature for at least an hour. Using a marking pen (ImmEdge Pen H-4000; PAP pen, VWR Laboratories), sections were circled. An appropriate amount of probe was pre-hybridized with a mixture of 1X salt solution (NaCl, Tris HCl, Tris Base, Na_2_HPO_4_.7H_2_0, NaH_2_PO_4_, and 0.5 M EDTA) containing 50% deionized formamide (Sigma), 10% dextran sulfate, 1 mg/mL tRNA, and 1X Denhart's (Fisher) at 70°C for 5 mins. Hybridization with DIG–labeled antisense probes was carried out at 65°C overnight. The following day, slides were taken through a series of washes in a solution of 1X SSC, 50% formamide and 0.1% Tween-20 at 65°C. Slides were brought to room temperature and washed extensively in MABT (100 mM Maleic acid, 150 mM NaCl, and 0.1% Tween-20) and incubated in a blocking solution (MABT, Goat serum and 10% milk) for at least 2 hours. Anti-DIG Fab fragments (pre-absorbed against zebrafish fin tissue) were used at 1∶5000 dilution and incubated overnight at 4°C. On day 3, slides were washed 4X in MABT (30 min each) followed by 2X short washes (5–10 min) with staining buffer (100 mM Tris, 9.5, 50 mM MgCl_2_, 100 mM NaCl, and 0.1% Tween20). Slides were then transferred to 10% polyvinyl alcohol (PVA; MW: 86,000) staining solution with NBT/BCIP stock solution (Roche) and allowed to develop overnight at 37°C. When a purple color started to appear on the sections, the reaction was stopped by washing the slides with PBST for at least 3 h and then stored in PBST at 4°C until imaging. Sections were mounted in 100% glycerol and examined on a Nikon Eclipse 80i microscope.

### Morpholino mediated gene knock-down in regenerating fins

All morpholinos (MOs) used in this study were fluorescein tagged, obtained from Gene Tools, LLC and used at a 1 mM concentration for injection. The sequences for MOs used in this study can be found in Table 1. Injection and electroporation were carried out as described previously [18]. Briefly, 3 dpa fish were anesthetized and approximately 50 nl of MO (targeting or mismatch [MM] control) was injected into either the dorsal or the ventral half of the tail fin. The un-injected half of the fin served as the internal control. Following injection, the entire fin was electroporated using a CUY21 Square Wave electroporator (Protech International Inc). MO-positive fish were selected 24 hpe (hours post electroporation) by examination under a fluorescence microscope. For H3P staining and qRT-PCR analysis, the fins were harvested 1 dpe, and for the analysis of regenerate length and segment length, the fins were harvested 4 dpe. For each MO (i.e., targeting or mismatch) 5–6 fish were injected on one half of the fin with the un-injected side serving as an internal control. Reproducibility was confirmed by testing the MO in three independent experiments. Statistical significance was determined using the student's *t*-test (P<0.05).

### Immunochemistry and detection of HA

The following primary antibodies were used: Rabbit anti-histone-3-phosphate (anti-H3P, Millipore, 1∶200); Mouse anti-Hapln1a antibody (MD Bioproducts, 1∶500). The following secondary antibodies were used: anti-mouse Alexa 488 or 546 (1∶200); anti-rabbit Alex 546 (1∶200). For H3P staining, whole fins were harvested 1 dpe, after MO mediated knock-down and the experiment was carried out as described [18]. HA was detected by biotinylated-HABP (US Biological, 1∶300) followed by fluorescently labeled streptavidin-Alexa-546 conjugate, (Invitrogen, 1∶200). For detection of Hapln1a or HA after MO injection (targeting or MM control), the fins were harvested 1 dpe, fixed in 4% PFA overnight and then stored in Methanol. Cryosectioning was done as described [18] and the sections were allowed to dry overnight. The sections were then rehydrated in PBS, twice for 10 min followed by two washes with Immunostaining block (2% BSA, 0.1% Tween in PBS). Then, sections were blocked for another 1 h at room temperature with the immunostaining block and then incubated in primary antibody or biotin-HABP overnight at 4°C. The sections were washed with block (3x, 15 min each), incubated at room temperature for 1 hr with secondary antibody or streptavidin-Alexa-546 (pre-absorbed for 1 hr. at room temperature with fixed zebrafish fins to reduce background staining), washed (3x, 15 min each), incubated with HOECHST stain for 10 min at room temperature, followed by a quick wash with distilled water. They were blotted dry and then mounted for imaging. To test for specificity of HABP, WT fin sections were treated with bovine hyaluronidase (Sigma-H3506, final concentration 200 U/ml) in 10 mM phosphate buffer containing 100 mM NaCl (pH 6.0) for 2 hours at 37°C [33], [34]. Untreated sections were incubated in buffer without enzyme. Following treatment the sections were washed two times in Immunostaining block before staining for HABP as described above.

### Lysate preparation and Immunoblotting

For WT and *sof ^b123^*, 5 dpa regenerating fins were harvested from 10–15 fish. For MO injected fins, the injections were performed at 3 dpa fish (20–25 fish) and harvested the next day. Regenerating fins were harvested into 300–500 µl of RIPA lysis buffer, supplemented with protease inhibitor (Thermo scientific, Halt™ Protease and Phosphatase Inhibitor Cocktail) and homogenized using a tissue homogenizer (Bio-Gen, PRO 200) at high speed (5X) for 5 seconds with 10 second cooling intervals. Homogenized samples were centrifuged at 200 g for 10 min at 4°C and supernatant was used for further analysis. The protein samples were concentrated using a lyophilizer (Edwards-Freeze dryer super modulyo) and the pellet was resuspended in a minimal volume of PBS containing (Thermo scientific, Halt™ Protease and Phosphatase Inhibitor Cocktail, 100X) and the protein levels estimated by Bradford's assay.

To release Hapln1a from HA for detection by immunoblotting, equal concentrations of Hapln1a MO and MM lysates were treated with 100 U/ml of bovine hyaluronidase (Sigma) for 2 hours at 37°C as described [35]. Immunoblotting was performed as described [13] using mouse anti-Hapln1a (MD Bioproducts, 1∶500) and anti-α-tubulin (Sigma, 1∶3000) in combination with peroxidase-conjugated goat anti-mouse IgG (1∶10,000). Signal detection was performed using ECL Prime western blotting detection reagent (Amersham™ - GE Healthcare).

### Measurements

The regenerate length, segment length, and the number of dividing cells were estimated/calculated as described [18]. For each experiment, at least 5 fish were evaluated in triplicate and a student's *t*-test was performed to evaluate statistical significance. Image Pro software was used to measure the regenerate length from the plane of amputation to the tip of the fin and segment length between two joints under bright field. Cell number was calculated by counting the number of histone-3-phosphate (H3P) positive cells from the distal-most 250 µm^2^ of the 3^rd^ fin ray.

### Quantitative real-time PCR

For qRT-PCR analysis, TRIZOL RNA extraction was made from the 5 dpa regenerating fins of wild-type, *sof ^b123^*, and *alf ^dty86^* and 1 dpe for MO injected fins (targeting or MM). A minimum of 10 fins was used for total RNA extraction. For each sample, 1 µg of total RNA were reverse transcribed with SuperScript III reverse transcriptase (Invitrogen) using oligo-dT primers. Primers for qPCR analysis of *hapln1a, sema3d* and *actin* were designed using Primer express software (Table 1). Three independent RNA samples were used for the experimental comparison and qPCR for each gene was done in duplicates. The samples were analyzed using Rotor-Gene 6000 series software (Corbette Research) and the average cycle number (C_T_) was determined for each amplicon. Delta C_T_ (ΔC_T_) values represent normalized expression levels of the test with respect to *actin*, the internal control. The relative level of gene expression, which is the fold difference, was determined using the delta delta C_T_ (ΔΔC_T_) method (i.e., 2^−ΔΔCT^).

## Results

### Hapln1a is expressed downstream of cx43

**Figure 1:**
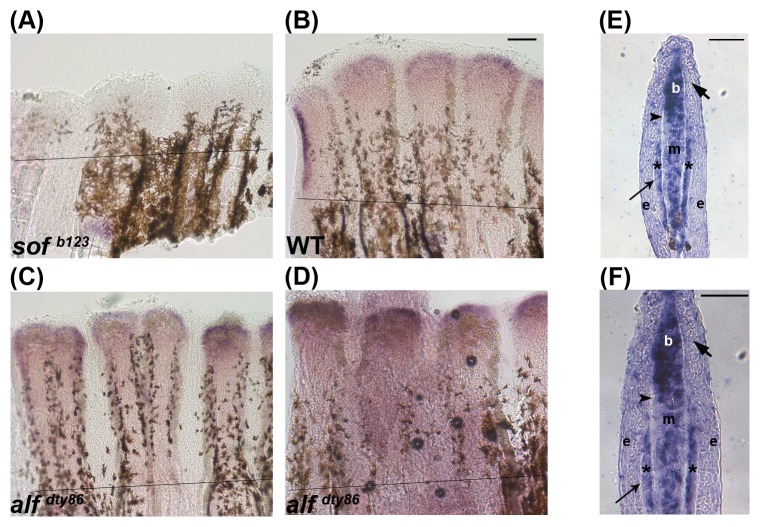
Validation of microarray results by in situ hybridization using whole mount in situ hybridization on 5 dpa regenerating fins. (A) *hapln1a* expression is reduced in *sof ^b123^* compared with WT (B). *alf ^dty86^* expression is variable (C,D). (E,F) In situ hybridization on a WT 5 dpa cryosection reveals compartment specific expression of *hapln1a*, mainly in the blastema (b), in the mesenchyme (m), and skeletal precursor cells (*). The thick arrow identifies the basal layer of the epidermis, which underlies the epidermis (e). The thin arrow identifies lepidotrichia and the arrowhead identifies the actinotrichia. The amputation plane is indicated in panels A-D. Scale bar in E and F is 50 µm.

Recently, we described a microarray strategy to identify genes that function downstream of Cx43. We identified ∼50 genes that were both downregulated in *sof ^b123^* and upregulated in *alf ^dty86^* [18]. One of the candidate genes identified was *hapln1a*. To validate that *hapln1a* is expressed downstream of *cx43*, we compared the expression levels of *hapln1a* in WT, *sof ^b123^*, and *alf ^dty86^* regenerating fins by whole mount in situ hybridization (Figure 1). As expected, *hapln1a* mRNA expression appeared to be downregulated in *sof ^b123^*. We noticed some variability among *alf ^dty86^* regenerating fins, where some fins exhibited upregulation and some appeared more similar to WT regenerating fins (two representative images are shown in Figure 1). Therefore, we performed quantitative RT-PCR (qPCR) to evaluate the level of *hapln1a* expression in *sof ^b123^* and *alf ^dty86^* regenerating fins compared with WT (Table 2). As expected, *hapln1a* is downregulated in *sof ^b123^*. However, we did not observe an upregulation of *hapln1a* in *alf ^dty86^*, suggesting that among a population of *alf ^dty86^* regenerating fins, there is little difference in the expression level of *hapln1a* between WT and *alf ^dty86^*.To determine the tissue-specific expression of *hapln1a,* we performed in situ hybridization on cryosections. The longitudinal section of a regenerating fin ray reveals several outer epidermal layers, including the basal layer of epidermis, which is adjacent to the mesenchyme. The mesenchyme is located medially. Within the mesenchymal compartment, a blastema is established at the distal, or growing end, of each ray. Blastemas are comprised largely of dividing cells that contribute to new tissue growth (reviewed in [36], [37]). The *hapln1a* mRNA is expressed throughout the mesenchyme with slightly higher expression in the blastema, and to a lesser extent in the skeletal precursor cells (Figure 1).

**Table 2:** Quantitative RT-PCR confirms changes in gene expression.

Samples compared	Gene	ΔC_T(Mut/MO)_ Gene-Actin^a^	ΔC_T (WT/MM)_ Gene-Actin^a^	ΔΔC_T_ = ΔC_T((Mut/MO) – (WT/MM))_^b^	Fold difference relative to WT/MM ^c^
***sof ^b123^*** ** with WT**	*hapln1a*	6.51±0.13	4.99±0.10	1.52±0.13	0.35 (0.32–0.38)
***alf ^dty86^*** ** with WT**	*hapln1a*	4.97±0.25	4.99±0.10	−0.02±0.25	1.01 (0.85–1.20)
***cx43*** ** KD with MM**	*hapln1a*	6.57±0.23	5.67±0.08	0.90±0.23	0.53 (0.46–0.63)
***sema3d*** ** KD with MM**	*hapln1a*	4.89±0.20	5.13±0.19	−0.24±0.20	1.18 (1.03–1.36)
***hapln1a*** ** KD with MM**	*cx43*	2.78±0.10	2.84±0.19	−0.06±0.10	1.04 (0.97–1.12)
***hapln1a*** ** KD with MM**	*sema3d*	6.69±0.15	6.90±0.15	−0.21±0.15	1.15 (1.04–1.28)

a. The ΔC_T_ value is determined by subtracting the average Actin C_T_ value from the average Gene C_T_ value. The standard deviation of the difference is calculated from the standard deviations of the gene and actin values using the Comparative Method.

b. The calculation of ΔΔC_T_ involves subtraction by the ΔC_T_ calibrator value. This is subtraction of an arbitrary constant, so the standard deviation of ΔΔC_T_ is the same as the standard deviation of the ΔC_T_ value.

c. The range given for gene relative to WT/MM is determined by evaluating the expression: 2∧ –ΔΔC_T_ with ΔΔC_T_ + s and ΔΔC_T_ – s, where s  =  the standard deviation of the ΔΔC_T_ value.

In order to provide a secondary test to demonstrate *cx43*-dependence of *hapln1a*, we examined its expression in fins treated for *cx43* knock-down [13]. Indeed, the expression of *hapln1a* is reduced in WT fins treated for *cx43* knock-down compared to *cx43* mismatch-treated fins by both in situ hybridization (Figure S1) and by qPCR (Table 2). In contrast, *cx43* expression is not reduced in WT fins treated for *hapln1a* knock-down (Table 2), indicating that *cx43* expression does not depend on *hapln1a*. In summary, we find that *hapln1a* is reduced both in *sof ^b123^* and in *cx43* knock-down fins, providing independent confirmation that *hapln1a* is expressed in a *cx43*-dependent manner. Together, these data support the conclusion that *hapln1a* is molecularly downstream of *cx43*.

**Figure 2:**
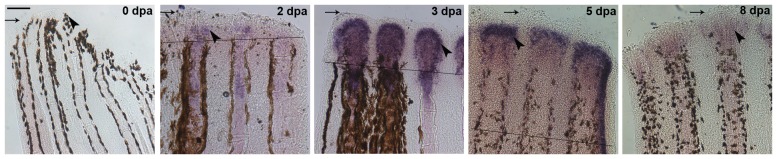
*hapln1a* expression pattern at different time points in regenerating fin. Whole mount in situ hybridization for *hapln1a* on regenerating fins at different time points. The *hapln1a* gene is not expressed during normal fin growth (uncut). Expression of *hapln1a* initiates around 2 dpa and is maximally expressed at 3 dpa, followed by gradual reduction at 5 dpa and 8 dpa. Arrow identifies the distal end of the fin; the arrowhead identifies region of staining (or where staining would be observed in uncut fins). The amputation plane is indicated for 2 dpa, 3 dpa, and 5 dpa, and is out of the field of view for the 8 dpa image. Scale bar is 50 µm.

To evaluate when during fin growth *hapln1a* may be required in WT regenerating fins, we examined the expression pattern of *hapln1a* in non-regenerating fins, and at 2 dpa, 3 dpa, 5 dpa and 8 dpa (Figure 2). In non-regenerating fins *hapln1a* expression was not detectable. By 2 dpa, the expression of *hapln1a* was slightly up-regulated, and by 3 dpa *hapln1a* was expressed strongly within each fin ray. At 5 dpa, there was a clear decrease in expression, and its expression was decreased even more by 8 dpa. Thus, *hapln1a* expression is expressed at 2 dpa and is maximally expressed at 3 dpa. The maximum expression at 3 dpa corresponds with the time point at which maximum rate of regeneration has been observed [38].

### Hapln1a knock-down is functionally downstream of Cx43

**Figure 3:**
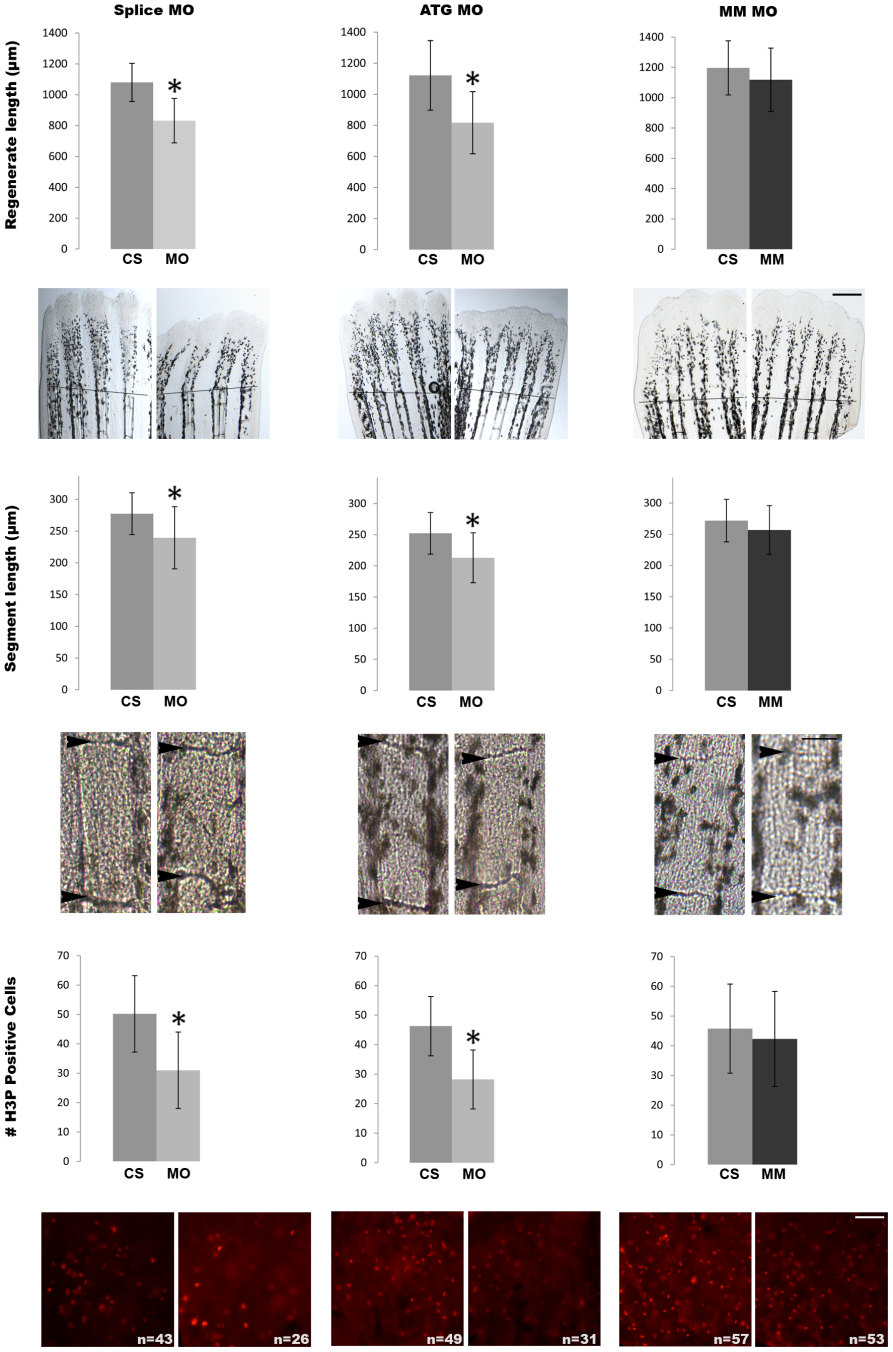
Morpholino mediated knock-down of *hapln1a* recapitulates all *cx43*-dependent phenotypes. (Top row) Total regenerate length was measured in MO and MM treated fins. Hapln1a knock-down resulted in reduced fin length (*). The dotted line represents the amputation plane. (Middle row) Segment length was measured in MO and MM treated fins. Hapln1a knock-down resulted in reduction of segment length (*). Arrowheads identify joints. (Bottom row) Total number of H3P positive cells (red) was counted. Images of representative whole fins stained for H3P are shown for each condition. Knock-down of Hapln1a resulted in reduced cell proliferation (*) compared to the mismatch control. MO represents targeting morpholino; ATG MO targets translation initiation; Splice MO targets splicing event; MM represents control morpholino with 5 mismatches to target sequence; CS represents uninjected/untreated side. All experiments were completed in triplicate with a minimum of five fins treated with MO per trial. Statistical significance was determined by student's *t*-test where p<0.05. By student's *t*-test, there is no statistical difference between MM and CS. Error bars represent the standard deviation. Scale bar is 50 µm.

In order to determine if *hapln1a* is functionally downstream of *cx43*, we completed morpholino-mediated gene knock-down of *hapln1a* in WT regenerating fins [13], [15]. Two targeting morpholinos (MOs) were generated, an ATG blocker (ATG MO) that inhibits protein translation and a splice blocker (Splice MO) that was designed to inhibit the splicing of intron 1. As a control, we used a mismatch MO (MM MO), which includes five mismatches to the target sequence for the ATG-blocking MO (Table 1). All MOs are conjugated to fluorescein, permitting validation of cellular uptake. Following microinjection and electroporation, MO-positive fish were selected for fluorescein-positive cells at 24 hours post electroporation (hpe). Positive fins were either harvested for analysis of cell proliferation or permitted to regenerate for 4 additional days for evaluation of segment length and regenerate length. The effect of *hapln1a* knock-down on cell proliferation was evaluated by counting the number of mitotic cells detected by H3P immunostaining. Regenerate length was measured as the distance between the amputation plane and the distal end of the fin. Segment length was measured as the distance between the first two joints in the regenerate. Interestingly, we found that *hapln1a* knock-down with both the ATG MO and Splice MO exhibited the same phenotypes as *cx43* knock-down. Thus, knock-down fins exhibited reduced fin length (Figure 3A), reduced segment length (Figure 3B), and reduced cell proliferation (Figure 3C). The specificity of these knock-downs is demonstrated by the use of two independent gene-targeting MOs and also by the mismatch control. Collectively, these data reveal that *cx43* and *hapln1a* act in a common pathway to promote cell proliferation and to inhibit joint formation.

### Hapln1a knock-down destabilizes HA

**Figure 4:**
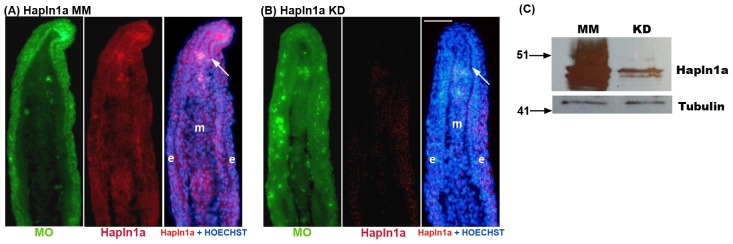
Morpholino mediated knock-down of *hapln1a* in WT regenerating fins results in reduced Hapln1a. Immunostaining for Hapln1a and HOECHST staining for DNA (blue). The green reveals the location of the targeting and control MOs, which are fluorescein tagged. (A) Longitudinal section of a fin ray treated with Hapln1a control morpholino (MM). (B) Longitudinal section of a fin ray knocked down for Hapln1a with a targeting morpholino (KD). Compared to the control MM fins, Hapln1a knock-down (KD) fins exhibit reduced staining for Hapln1a. (C) Immunoblots confirming reduced levels of Hapln1a protein following Hapln1a knockdown. Hapln1a-MO treated fins (KD) were compared to control morpholino (MM). Tubulin was used as a loading control. Arrow identifies the basal layer of the epidermis; m, mesenchyme; e, epithelium. Scale bar is 50 µm.

**Figure 5:**
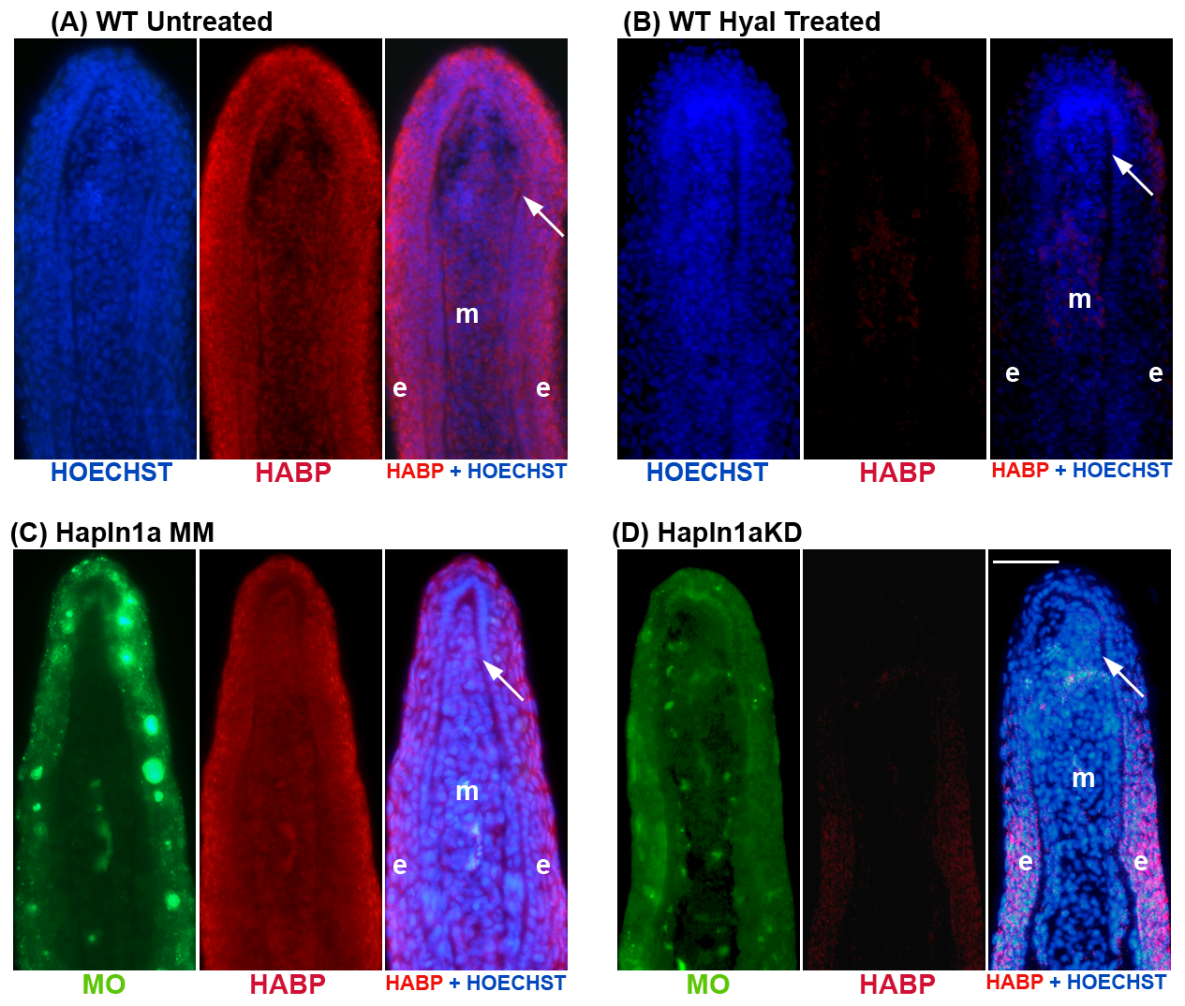
Morpholino mediated knock-down of *hapln1a* in WT regenerating fins results in reduced HA levels. HA is detected by biotin-HABP with streptavidin-Alexa-546. HOECHST detects DNA (blue). The green reveals the location of the targeting and control MOs, which are fluorescein tagged. (A,B) Fin sections from 5 dpa WT regenerating fins were either untreated or treated with hyaluronidase. The reduced signal in the hyaluronidase-treated sections demonstrates that HABP detects HA. (C) Longitudinal section of a fin ray treated with Hapln1a control morpholino (MM). (D) Longitudinal section of a fin ray knocked down for Hapln1a with a targeting morpholino (KD). Compared to the control MM fins, Hapln1a knock-down (KD) fins exhibit reduced staining for HA. Arrow identifies the basal layer of the epidermis; m, mesenchyme; e, epithelium. Scale bar is 50 µm.

To evaluate the effect of *hapln1a* knock-down on HA in WT regenerating fins, we completed a MO-mediated *hapln1a* knock-down (i.e. using the ATG MO) and evaluated both Hapln1a staining and HA staining on the treated fins. The success of *hapln1a* MO-mediated protein knock-down was further confirmed by immunostaining. Thus, comparison of the MM MO treated fins with the ATG MO treated fins revealed that Hapln1a protein is reduced following *hapln1a* knock-down (Figure 4A-B). Moreover, reduced Hapln1a protein levels in ATG MO treated fins was confirmed through immunoblotting (Figure 4C). Hapln1a protein is glycosylated and typically detected as several bands above its predicted molecular weight of 38 kD [35]. All bands are reduced following Hapln1a knock-down. Since Hapln1a is crucial for the stabilization of the HA-PG network in the ECM, we also evaluated the level of HA in *hapln1a* knock-down fins. We utilized biotinylated HA-binding protein (HABP-biotin) in combination with streptavidin-Alexa546 to detect HA. First, we demonstrate that HABP is specific for the detection of HA by treating fin sections with the HA-degrading enzyme hyaluronidase. Endogenous levels of HA may be observed in WT 5 dpa fins (untreated). In contrast, treatment of fin sections with hyaluronidase greatly reduced the staining by HABP (Figure 5A-B), suggesting that HA is required for detection. Importantly, we also observed a clear reduction in the amount of detectable HA following *hapln1a* knock-down (Figure 5C-D). These findings strongly support the hypothesis that HA is destabilized in the absence of sufficient Hapln1a.

**Figure 6:**
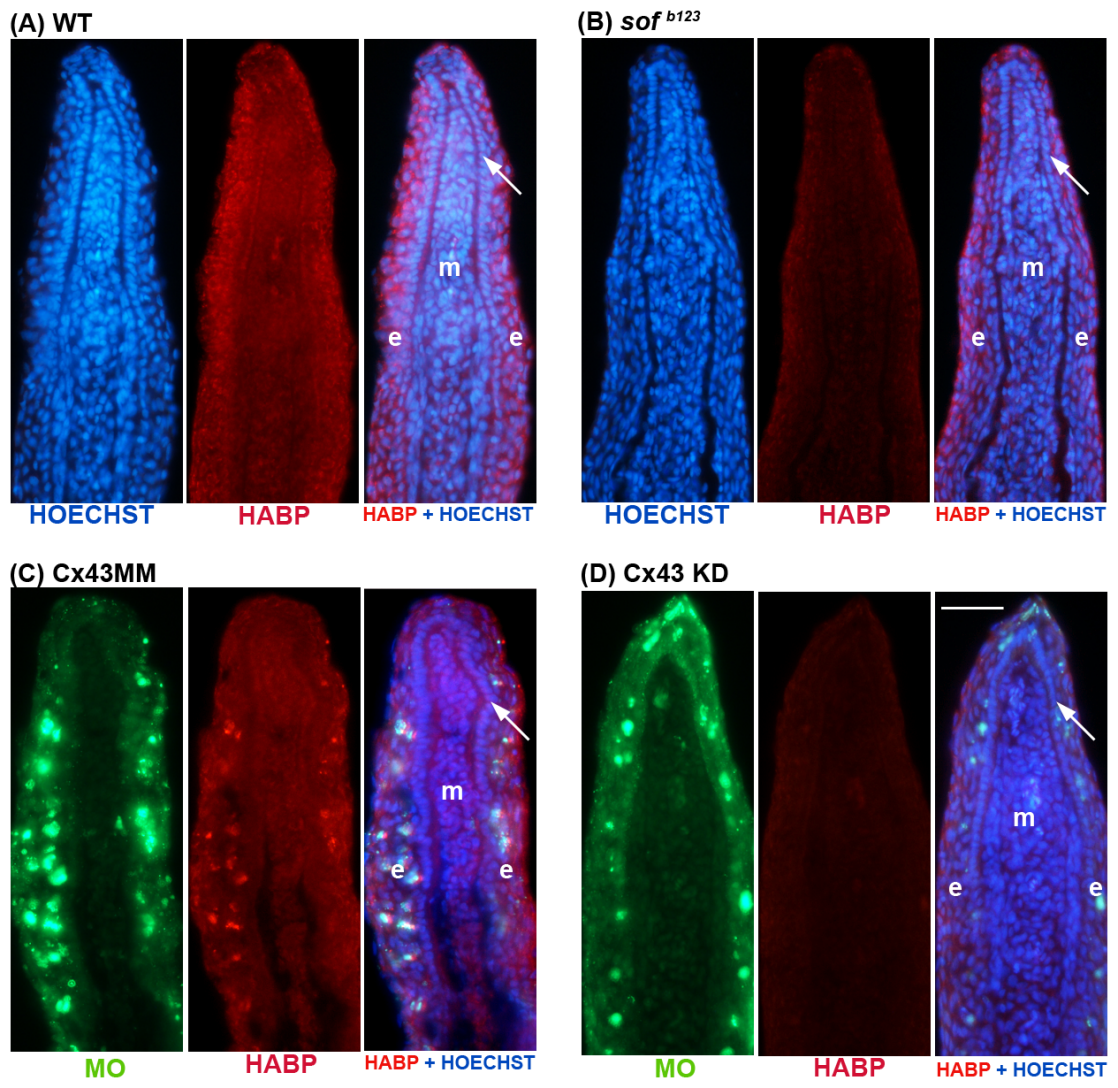
HA levels are reduced in *sof ^b123^* and in *cx43*-KD regenerating fins. Immunostaining for HA (Red) and HOECHST staining for DNA (blue). In the panels treated for *cx43*-MO or MM, the green reveals the location of the MOs, which are fluroescein tagged. HA levels were detected as described in Figure 4. (A,C) HA levels in WT 5 dpa regenerating fins and in WT fins treated for *cx43*-MM. (B,D) HA levels are reduced in *sof ^b123^* regenerating fins and in WT fins treated for *cx43*-MO. Arrow identifies the basal layer of the epidermis; m, mesenchyme; e, epithelium. Scale bar is 50 µm.

To further demonstrate that *hapln1a* functions in a *cx43*-dependent manner, we next evaluated HA levels in both *sof ^b123^* regenerating fins and in fins treated for *cx43*-knockdown [13]. Since *hapln1a* levels depend upon Cx43 activity, we predicted that reduced Cx43 would similarly lead to reduced levels of HA. Indeed, this is what we found. In *sof ^b123^* regenerating fins compared with WT regenerating fins, we observed reduced levels of HA (Figure 6A-B). Similarly, in WT fins treated for MO mediated *cx43* knockdown, HA levels are reduced compared with the MM control-treated fins (Figure 6C-D). Together with our previous findings, these data strongly support the conclusion that *hapln1a* functions in a common pathway with *cx43*, and that this pathway mediates cell proliferation and joint formation at least in part through influencing the Hapln1a-based ECM.

### Hapln1a functions in a cx43-dependent and sema3d-independent genetic pathway

**Figure 7:**
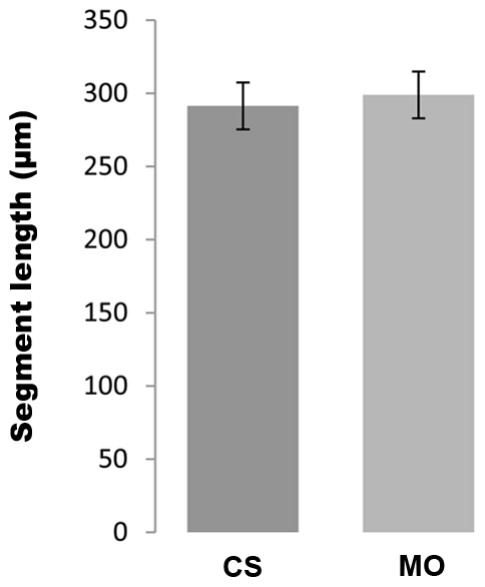
Hapln1a knock-down does not rescue segment length in *alf ^dty86^*. Compared to the uninjected control side (CS) the Hapln1a morpholino treated side (MO) does not show significant difference in the segment length. Statistical significance was determined by Student's *t*-test where p<0.05. Error bars represent the standard deviation.

In our recent study, we found that Cx43 and Sema3d function in a common molecular pathway to promote cell proliferation and joint formation [18]. We next wished to determine how *hapln1a* contributes to this previously defined pathway. Our prior findings suggest that Sema3d utilizes the Nrp2a receptor to mediate cell proliferation and the PlxnA3 receptor to mediate joint formation. Indeed, in addition to Cx43 knock-down, both Sema3d- and PlxnA3 knock-down rescues joint formation in *alf ^dty86^*. Therefore, we first tested if *hapln1a* knock-down functioned similarly. Interestingly, segment length was not rescued in *alf ^dty86^* fins by *hapln1a* knock-down (Figure 7). Failure of *hapln1a* knock-down to rescue segment length in *alf ^dty86^* was not due to a failure of the knock-down since both Hapln1a and HA were similarly reduced (i.e. evaluated by immunostaining for Hapln1a protein or for HA levels as completed above, see Figure S2). The finding that *hapln1a* knock-down does not rescue joint formation in *alf ^dty86^* is consistent with our finding that *hapln1a* is not up-regulated in *alf ^dty86^*. Moreover, this suggests that *hapln1a* functions independently of *sema3d*-*plxna3*-dependent joint formation. We next evaluated changes in *hapln1a* and *sema3d* gene expression following reciprocal knock-down experiments. Thus, in *sema3d* knock-down fins *hapln1a* is not affected (Table 2), suggesting that *hapln1a* is not downstream of *sema3d*. Similarly, in *hapln1a* knock-down fins *sema3d* expression is not affected (Table 2), suggesting that *sema3d* is not downstream of *hapln1a*. Collectively, our findings provide evidence that *hapln1a* and *sema3d* both function downstream of *cx43*, but independently of each other. Therefore, these data suggest that at least two pathways contribute to *cx43*-dependent cell proliferation and *cx43*-dependent joint formation (Figure 7A). Future studies will be completed to determine if the Sema3d and Hapln1a gene products instead functionally interact in a common pathway.

## Discussion

**Figure 8:**
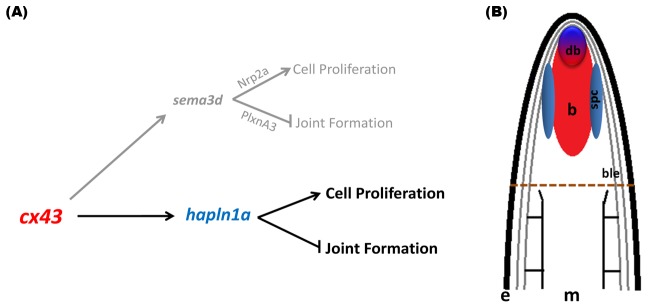
Model depicting Cx43-Hapln1a mediated effect on skeletal patterning during fin regeneration. (A) Proposed pathway for Hapln1a mediated effects of Cx43. Hapln1a functions in a *cx43*-dependent, but *sema3d*-independent pathway, positively influencing cell proliferation and inhibiting joint formation. (B) Cartoon illustrating the compartments of gene in the regenerating fin: The *cx43* mRNA is expressed throughout the mesenchyme (red) accompanied by *cx43*-dependent *hapln1a* upregulation in the same compartment (red), but primarily in the distal blastema (blue-red) and to a lesser extent in the proximal skeletal precursor cells (blue). e, epithelium; m, mesenchyme; b, blastema; db, distal blastema; spc, skeletal precursor cells; ble, basal layer of epidermis.

This is the first report to provide a functional analysis of *hapln1a* in zebrafish. Our finding that Hapln1a functions downstream of Cx43 is supported by multiple independent lines of evidence. The *hapln1a* gene exhibited reduced expression levels in *sof ^b123^* and *cx43* knock-down fins compared to WT regenerating fins by in situ hybridization. Additionally, qPCR analysis confirmed that *hapln1a* expression is dependent upon the level of *cx43* expression. Knock-down of Hapln1a using two independent MOs recapitulated all of the Cx43-dependent phenotypes, namely reduced regenerate length, reduced segment length, and reduced cell proliferation. Thus, *hapln1a* functions downstream of *cx43*. Moreover, we found that HA levels were strongly reduced in Hapln1a knock-down fins, suggesting that the loss of Hapln1a protein influences the stability of HA. The finding that HA levels are similarly reduced in *sof ^b123^* and in *cx43* knock-down fins provides additional evidence that *cx43* and *hapln1a* function in a common pathway. Attempts to include *hapln1a* in the established *cx43*-*sema3d* pathway suggest that *hapln1a* may function independently of *sema3d* (Figure 8A). Therefore, Cx43 promotes cell proliferation and suppresses joint formation via the coordination of (at least) two downstream pathways. We find it interesting that Hapln1a is located in the ECM that physically connects the medial *cx43*-positive compartment of dividing cells [8] and the lateral *sema3d*-positive compartment of skeletal precursor cells [18]. The functional significance of this observation is unclear.

Hapln1a belongs to the hyaluronectin superfamily, which includes four members in mammals (HAPLNs1-4) and five in zebrafish, where the *hapln1* gene appears to have duplicated into *hapln1a* and *hapln1b*. These genes share high degrees of sequence homology, especially in the amino acid sequence coding for the link module [25], [39]. Hapln1 was first identified in cartilage, however it can also be found in noncartilaginous tissue like sclera [40], aorta [41], brain [42], dermis of the embryonic skin [43], and in chicken embryonic mesonephros [44]. During zebrafish embryogenesis, expression of *hapln1a* has been observed in multiple tissues, including somites, floor plate, hypochord, and rhombomeres [45]. During jaw and skull formation, *hapln1a* expression co-localized with the PG aggrecan in the pharyngeal arches and with the PG dermacan in the pectoral fins [45]. Functional studies were not performed as part of these studies. However, reduced Hapln1 function is correlated with skeletal defects in other animals, including human. For example, single-nucleotide polymorphisms identified in the *HAPLN1* gene have recently been associated with spinal osteoarthritis in aging female populations [46]. Moreover, targeted gene knockout of *CRTL1* (aka *HAPLN1*) in the mouse reveals that Crtl1/Hapln1 is indispensible during skeletal development. For example, the *CRTL1^−/−^* mouse showed defects in cartilage and bone development with short limbs and craniofacial abnormalities, suggesting a prominent function for Crtl1 during chondrocyte differentiation [19]. In addition to these skeletal defects, *CRTL1^−/−^* mice die perinatally and exhibit a spectrum of myocardial defects. These defects have been attributed to the reduction in the PG versican [47], which may promote cell proliferation [48]–[50]. Interestingly, cartilage-specific expression of transgenic Crtl1/Hapln1a inhibits perinatal lethality and rescues skeletal abnormalities in *CRTL1^−/−^* mice [51], reinforcing the importance of the HA-PG network during heart development and skeletogenesis. These studies provide evidence that the “link” function of Hapln1 is critical for stability of components of the ECM and for regulation of cell differentiation and cell proliferation. Our findings on the function of Hapln1a in the regenerating fin are consistent with the role of Hapln1 in the developing mouse skeleton. Future studies will be focused on defining how Hapln1a mediates skeletal growth and patterning.

We propose that Cx43 activity in the blastema activates gene expression of *hapln1a* in the medial mesenchyme (Figure 8B). Secretion of Hapln1a in the ECM establishes the HA-PG network, stabilizes HA, and contributes to the regulation of cell proliferation and joint formation via unknown mechanisms. The Hapln1a-dependent ECM might be involved in providing the necessary microenvironment for the surrounding cells. Alternatively, Hapln1a may be required to maintain a stable population of HA, which in turn mediates signaling pathways through interaction with HA-specific cell surface receptors. Indeed, it is known that HA has diverse functions in skeletal biology including bone remodeling [22], bone resorption [52], and osteogenesis [53], [54]. Further, there is growing evidence that the ECM can influence interactions between locally secreted growth factors and their receptors [55]–[59]. Continued studies are required to determine if Hapln1a plays direct or indirect roles in influencing cellular behaviors required during fin skeletal morphogenesis.

## Conclusions

The identification of the Cx43-dependent Hapln1a pathway is novel and reveals tangible roles for the ECM during bone growth and skeletal patterning. We find that reduced Hapln1a levels are correlated with reduced HA levels, which may provide insights into the underlying mechanism of Hapln1a function. Combined with known skeletal defects associated with the loss of Hapln1 in the mouse, and with skeletal diseases associated with Hapln1 polymorphisms in human, these findings demonstrate that the role of Hapln1a is conserved in zebrafish. Therefore, continued studies designed to elucidate the mechanism of Hapln1a-dependent cell proliferation and joint formation will provide new and relevant insights into skeletal development in all vertebrates.

## Supporting Information

Figure S1 **Cx43 knock down results in reduced expression levels of** ***hapln1a*** **.** Whole mount in situ hybridization shows reduced expression of *hapln1a* in WT fins treated for *cx43* knock-down (Cx43KD) fins compared to WT fins treated for *cx43* mismatch control (Cx43MM) control fins.
(TIF)

Figure S2 **The** ***hapln1a*** **-KD was effective in** ***alf ^dty86^*** **.** Immunostaining for Hapln1a or HA (Red) and HOECHST staining for DNA (blue). The green reveals the location of the targeting and control MOs, which are fluroescein tagged (A) Longitudinal section of an *alf ^dty86^* fin ray treated with Hapln1a control morpholino (MM). (B) Longitudinal section of an *alf ^dty86^* fin ray knocked down for Hapln1a with a targeting morpholino (KD). Compared to the control MM fins, Hapln1a knock-down (KD) fins exhibit reduced staining for Hapln1a. HA was detected by biotinylated-HABP followed by streptavidin-Alexa 546 conjugate. (C) Longitudinal section of an *alf ^dty86^* fin ray treated with Hapln1a control morpholino (MM). (D) Longitudinal section of an *alf ^dty86^* fin ray knocked down for Hapln1a with a targeting morpholino (KD). Compared to the control MM fins, Hapln1a knock-down (KD) fins exhibit reduced staining for HA. Arrow identifies the basal layer of the epidermis; m, mesenchyme; e, epithelium. Scale bar is 50 µm.
(TIF)
