# Supplementary figures and images for: Hapln1a Is Required for Connexin43-Dependent Growth and Patterning in the Regenerating Fin Skeleton
Source: PLoS One. 2014 Feb 12;9(2):e88574. doi: 10.1371/journal.pone.0088574 (PMC3922931; doi:10.1371/journal.pone.0088574)

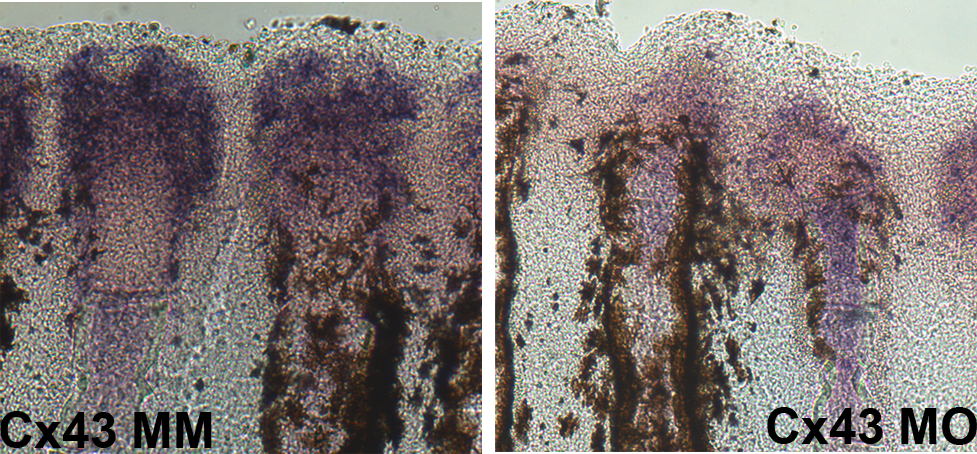

Supplement: Figure S1 — Cx43 knock down results in reduced expression levels of hapln1a . Whole mount in situ hybridization shows reduced expression of hapln1a in WT fins treated for cx43 knock-down (Cx43KD) fins compared to WT fins treated for cx43 mismatch control (Cx43MM) control fins. (TIF) [file pone.0088574.s001.tif]

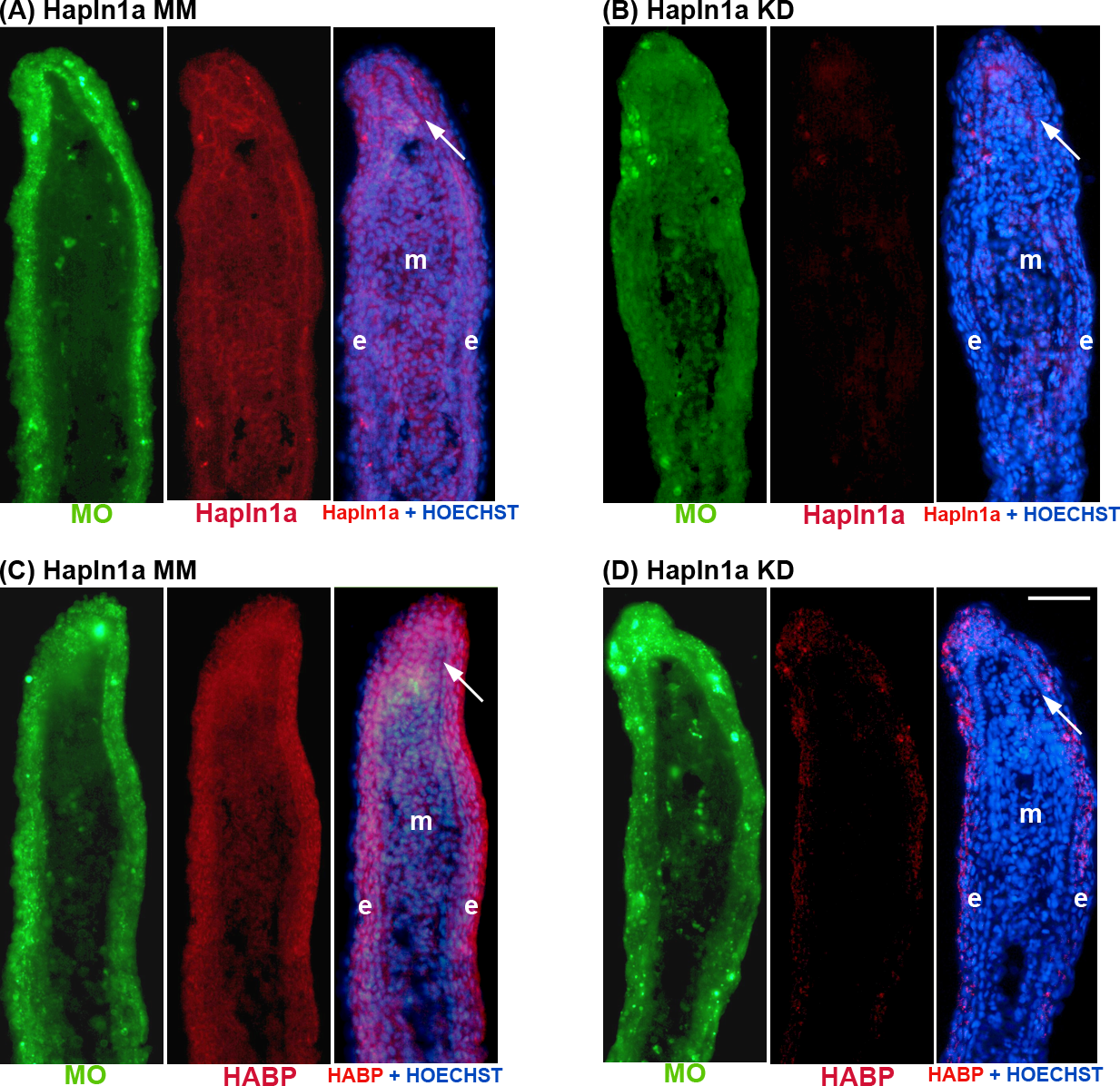

Supplement: Figure S2 — The hapln1a -KD was effective in alf dty86 . Immunostaining for Hapln1a or HA (Red) and HOECHST staining for DNA (blue). The green reveals the location of the targeting and control MOs, which are fluroescein tagged (A) Longitudinal section of an alf dty86 fin ray treated with Hapln1a control morpholino (MM). (B) Longitudinal section of an alf dty86 fin ray knocked down for Hapln1a with a targeting morpholino (KD). Compared to the control MM fins, Hapln1a knock-down (KD) fins exhibit reduced staining for Hapln1a. HA was detected by biotinylated-HABP followed by streptavidin-Alexa 546 conjugate. (C) Longitudinal section of an alf dty86 fin ray treated with Hapln1a control morpholino (MM). (D) Longitudinal section of an alf dty86 fin ray knocked down for Hapln1a with a targeting morpholino (KD). Compared to the control MM fins, Hapln1a knock-down (KD) fins exhibit reduced staining for HA. Arrow identifies the basal layer of the epidermis; m, mesenchyme; e, epithelium. Scale bar is 50 µm. (TIF) [file pone.0088574.s002.tif]
